# Nonsegmental Vitiligo and Autoimmune Mechanism

**DOI:** 10.1155/2011/518090

**Published:** 2011-07-26

**Authors:** Naoki Oiso, Tamio Suzuki, Kazuyoshi Fukai, Ichiro Katayama, Akira Kawada

**Affiliations:** ^1^Department of Dermatology, Kinki University Faculty of Medicine, 377-2 Ohno-Higashi, Osaka-Sayama, Osaka 589-8511, Japan; ^2^Department of Dermatology, Yamagata University School of Medicine, Yamagata 990-9585, Japan; ^3^Department of Dermatology, Osaka City University Graduate School of Medicine, Osaka 545-8585, Japan; ^4^Department of Dermatology, Osaka University Graduate School of Medicine, Osaka 565-0871, Japan

## Abstract

Nonsegmental vitiligo is a depigmented skin disorder showing acquired, progressive, and depigmented lesions of the skin, mucosa, and hair. It is believed to be caused mainly by the autoimmune loss of melanocytes from the involved areas. It is frequently associated with other autoimmune diseases, particularly autoimmune thyroid diseases including Hashimoto's thyroiditis and Graves' disease, rheumatoid arthritis, type 1 diabetes, psoriasis, pernicious anemia, systemic lupus erythematosus, Addison's disease, and alopecia areata. This indicates the presence of genetically determined susceptibility to not only vitiligo but also to other autoimmune disorders. Here, we summarize current understanding of autoimmune pathogenesis in non-segmental vitiligo.

## 1. Nonsegmental Vitiligo

Nonsegmental vitiligo is acquired depigmented skin lesions mainly caused by autoimmune loss of melanocytes. Genetic and environmental factors are involved in the development. Recent genetic studies identified predisposed genes involving the development of nonsegmental vitiligo [1–5]. Current histopathological studies showed increased infiltration of dendritic cells, Th17 cells [6], and CD8+ cytotoxic T lymphocytes [7–10] in the margin of vitiligo and the reduced number of regulatory T (Treg) cells in the affected skin [11, 12].

## 2. Genetics in Nonsegmental Vitiligo

Genome-wide association studies are applied for identifying the candidate genes in multifactor-associated disorders such as nonsegmental vitiligo [1–5]. In 2007, NALP1 was revealed to be associated with the risk of nonsegmental vitiligo in Caucasians [1]. The subsequent studies in Caucasians identified multiple loci on major-histocompatibility-complex (MHC) class I molecules, MHC class II molecules, *PTPN22*, *LPP*, *IL2RA*, *UBASH3A*, *C1QTNF6*, *RERE*, *GZMB*, *TYR*, *FOXP1*, *CCR6*, *TSLP*, *XBP1*, and *FOXP3* [2–4]. Another study in Chinese Han population detected two independent loci within the MHC region and a locus at 6q27 containing *RNASET2*, *FGFR1OP*, and *CCR6* [5]. The current candidate genes of nonsegmental vitiligo are summarized in Table 1 [13–91]. These genes are classified into (i) autoantigen, (ii) innate immunity, (iii) innate and acquired immunity, and (iv) other function and miscellaneous. Importantly, nonsegmental vitiligo-susceptible genes are often involved in other autoimmune disorders. Further study is needed to identify additional nonsegmental vitiligo susceptible genes and to elucidate the pathologic mechanism of the genes in nonsegmental vitiligo.

## 3. Immunology in Nonsegmental Vitiligo

Nonsegmental vitiligo can be caused by an immunologically complex mechanism. A variety of melanocytes-expressing proteins have been identified as autoantigens. Cui et al. showed that 24 (83%) of 29 vitiligo patients had autoantibody to melanocytes-associated autoantigen versus 2 (7%) of 28 healthy controls [92]. Until now, various proteins have been detected as autoantigens including tyrosinase [93–95], tyrosinase-related protein 1 [96–99], tyrosinase-related protein 2 [96, 100], Pmel17 [101, 102], melanin-concentrating hormone receptor 1 [103], tyrosine hydroxylase [104], and lamin A [99]. Antityrosine hydroxylase autoantibody was more frequent in active vitiligo patients [104], suggesting potency as an activity marker. Using radioimmunoassay, Waterman et al. found positive antibody reactivity to gamma-enolase (8%), alpha-enolase (9%), heat-shock protein 90 (13%), osteopontin (4%), ubiquitin-conjugating enzyme (15%), translation-initiation factor 2 (6%), and Rab38 (guanosine-5′-triphosphate- (GTP-) binding protein) (15%) in nonsegmental vitiligo patient sera [105]. Melanocyte-specific antibodies might induce apoptosis of melanocytes [106]. Ruiz-Argüelles et al. reported that serum immunoglobulin G antibodies from vitiligo patients were able to penetrate cultured melanocytes *in vitro* and trigger apoptosis [106]. However, further investigations are required to elucidate the pathogenetic function of autoantibodies [107]. 

Histopathological studies demonstrated the increased dendritic cells [6], Th17 cells [6, 108], and CD8+ cytotoxic T lymphocytes [7–10] and the decreased naturally occurring CD4+CD25+FOXP3+ Treg cells [11, 12] at the margin of vitiligo lesions. The infiltrating cytotoxic CD8+ T cells recognize melanocyte-associated autoantigens and enable to locate at dermal-epidermal junctions [109]. The paucity of Treg in vitiligo skin causes perpetual antimelanocyte reactivity in nonsegmental vitiligo [110, 111]. The role of Treg and Th17 cells should be elucidated in order to understand the balance between the occurrence and suppression of the autoimmune reaction. 

The activation of inflammasome constructed by NOD-like receptors such as NALP-1 overproduces proinflammatory cytokines of IL-1*β* and IL-18, inducing apoptosis [112]. Interestingly, Wang et al. showed the increased IL-1*β* level in the vitiligo lesion and the expression of NALP-1 in the activated epidermal Langerhans cells and dermal dendritic cells [6]. As IL-1*β* is the essential cytokine to develop Th17 cells [6], IL-1*β* produced by activated inflammasome may involve the development of *nonsegmental* vitiligo.

## 4. Conclusion

Considerable progress is being made towards understanding the pathogenesis of nonsegmental vitiligo. Although a number of genes have been implicated by well-designed genome-wide association studies, we do not have good genotype-phenotype correlations. In the future, we can anticipate further advancement regarding specific interactions between disease-susceptible genes and gene-environment interactions.

##  Conflict of Interests

The authors declared that there is no conflict of interests.

## Figures and Tables

**Table 1:** Genes associated with nonsegmental vitiligo.

Gene	Function	Involved in other autoimmune diseases	Reference
(1) Autoantigen			
* TYR*	Production of melanin granules in melanosomes		
(2) Innate immunity			
* NALP1*	Inflammasome activation and release of proinflammatory cytokines such as IL-1*β* and IL-18	T1D, AD, CeD	[13–20]
* PTEN22*	A lymphoid tyrosine phosphatase of the protein-tyrosine phosphatase family controlling the signal from antigen receptors, costimulatory receptors, and cytokine receptors	GD, AA, RA, SLE, T1D, AD	[21–30]
(3) Adaptive immunity			
MHC	Major histocompatibility complex class I and II proteins being indispensable for antigen presentation	AT, AA, RA, SLE, T1D, PS	[31–43]
* FOXP3*	A functional marker for naturally occurring, thymus-selected CD4+CD25+FOXP3+ regulatory T cells	AT, T1D	[44–54]
* IL2RA*(CD25)	T-cell proliferation and a functional marker for naturally occurring, thymus-selected CD4+CD25+FOXP3+ regulatory T cells	GD, AA, RA, SLE, T1D, PS, PS,	[35, 51, 55–65]
* CCR6*	The CCR6/CCL20 chemokine receptor/ligand axis providing key homing signals recruiting both proinflammatory and suppressive T cell subsets	GD, RA	[55, 66–69]
* TSLP*	Induction of allogeneic naive T cells to differentiate into cytotoxic T cells, and induction of the proliferation and differentiation of CD4+CD8-CD25-thymocytes into CD4+CD25+FOXP3+ regulatory T cell		[70–72]
(4) Innate and adaptive immunity			
GZMB	(1) A family of conserved serine proteases stored within the cytotoxic granules of cytotoxic lymphocytes		[73, 74]
	(2) The formation of blebs containing various subcellular compartments on the surface of apoptotic cells		
	(3) Supply of these peptides for autoimmune response		
* XBP1*	Protective roles against oxidative stress, and unfolded protein response of a stress response to increased levels of unfolded proteins in the endoplasmic reticulum		[75–80]
(5) Other function and miscellaneous			
* LPP*	Involvement of cell-cell adhesion and cell motility	RA, T1D, CeD	[81–84]
* UBASH3A*	Involvement of growth factor withdrawal-induced apoptosis in T cells	RA, T1D, CeD	[85–89]
* C1QTNF6*	Unknown	RA, T1D, CeD	[90, 91]
* FOXP1*	A role in cardiac, lung, and lymphocyte development, and tumor-suppressive function		

AA: alopecia areata, AD: Addison's disease, AT: autoimmune thyroiditis, CeD: celiac disease, GD: Graves' disease, IL: interleukin, PS: psoriasis, PA: psoriatic arthritis, RA: rheumatoid arthritis, SLE: systemic lupus erythematosus, and T1D: type 1 diabetes.
